# A latent profile analysis of residents' knowledge, attitude, and practice toward common chronic diseases among ethnic minority area in China

**DOI:** 10.3389/fpubh.2022.940619

**Published:** 2022-07-25

**Authors:** Huaqin Hu, Yihua Xu, Yingshan Shao, Yaxin Liang, Qionghua Wang, Shunmei Luo, Heyun Lu, Heng Meng, Chenxi Liu

**Affiliations:** ^1^School of Public Health, Tongji Medical College, Huazhong University of Science and Technology, Wuhan, China; ^2^Lincang Second People's Hospital, Lincang, China; ^3^School of Medicine and Health Management, Tongji Medical College, Huazhong University of Science and Technology, Wuhan, China

**Keywords:** knowledge-attitude-practice (KAP), chronic disease, ethnic minorites, health literacy, latent profile analysis (LPA)

## Abstract

**Background:**

Health literacy plays an important role in preventing and managing chronic diseases, while low levels of health literacy among ethnic minorities are a major manifestation of health inequities. We believe that before effective health literacy intervention strategies, it is preferable to understand the features of health literacy among ethnic minorities. The present study firstly updated insights on health literacy among ethnic minorities by investigating the knowledge, attitude, and practice (KAP) profile of common chronic diseases in ethnic minority areas, and secondly discussed the KAP profiles in detail to inspire future health education interventions.

**Methods:**

A cross-sectional, health-literacy-sensitive study was conducted in China's typical ethnic minority area. Participants included 801 adult residents who lived in the ethnic minority area. The primary outcome was participant scores on the KAP questionnaire of common chronic diseases, followed by latent profile analysis to identify participants with similar KAP score patterns and determine whether membership in specific groups was associated with demographic or clinical characteristics.

**Results:**

The participants included 496 ethnic minorities (61.9%) and 305 Han Chinese (38.1%). Three-profile solution was determined after the latent profile analysis: incomplete transfer [I.T.] (*n* = 215), better practice [B.P.] (*n* = 301), and average [A.V.] (*n* = 285). IT group (26.84%) was characterized by the highest level of knowledge and attitude toward common chronic diseases and below average level for practice. Participants in B.P. group performed poorly in both knowledge and attitude toward common chronic diseases but had the highest level of practice. A.V. group reflected average knowledge, attitude, and practice toward common chronic diseases among three subgroups. Ethnic minorities were the dominant population in A.V. group (68.8%). Compared with other groups, the A.V. group contained the largest proportions of married participants (84.2%), participants with no formal education (46.7%), and high annual out-of-pocket medical expense (33.3%).

**Conclusion:**

A more specific and nuanced understanding of minority health literacy can enable service providers to provide more effective health education to their recipients, thereby improving health inequities.

## Introduction

Globally, chronic diseases have become one of the greatest threats to population health (1). Health illiteracy is the inability to understand and use medical information, which can affect access to and use of the health care system, and is a significant contributor to the burden of non-communicable diseases (2, 3). Conversely, as an indicator of an individual's ability to access, understand, assess, and use health information and make health-related decisions to maintain their health (4), health literacy plays an important role in the prevention and management of chronic diseases. Today, the differences in the distribution of health literacy among minority and mainstream populations have attracted the attention of researchers as a major manifestation of so-called health inequities (5).

The evidence supporting the role of health literacy in chronic disease prevention and management is based on health skills research (6). However, health literacy is not easily quantifiable, especially considering that “it (health literacy) is a dynamic construct that emerges from the interaction between patients/citizens and health care systems, organizations, and professionals” (7). Generally speaking, ethnic minority populations are at a low level of health literacy, and it is our concern to improve their health literacy through effective intervention strategies (8–10). Thus, it is a priority to understand the nature of their health literacy.

Despite the centrality of ethnic minorities' health literacy, empirical work on individual differences is nonexistent. At the individual level, knowledge, attitudes, and practice (KAP) toward common chronic diseases are critical components of health literacy (11). KAP theory itself is widely used in chronic disease health promotion. However, it is often used for post-intervention assessment rather than as a reference for pre-intervention design (12–14). The present study addresses this gap by investigating the profile of KAP regarding common chronic diseases among residents of a typical ethnic minority area. We will describe the diversity of these KAP profiles in detail to identify populations with similar KAP patterns and discuss their implications for future health literacy intervention as a work in progress.

## Methods

### Study design and participants

This study is a pilot study for a health literacy precise intervention study. The intervention study will be conducted in Linxiang District, Yunnan Province, Southwest China, and we divided the 10 townships in the district into ethnic minority areas (two townships) and non-ethnic minority areas (eight townships) using the percentage of ethnic minority population (21.88%) in the district in 2020 as the threshold. We will recruit adult participants aged 18 years or older in ethnic minority areas to implement a health literacy precise intervention based on latent profile analysis. In March 2021, we conducted a cross-sectional survey in Nami Township, one of the two ethnic minority areas. Participants were eligible if they: (1) were permanent residents (continuous cumulative residence in the area for at least 6 months) of Nanmei Township; (2) age 18 years or older; (3) voluntarily participate in the questionnaire and informed consent. Exclusion criteria: (1) age less than 18 years; (2) considered professionally unsuitable for the study (e.g., incapacity).

### Ethical consideration

Participants provided written informed consent and received a free physical examination for participation. The local hospital's ethics committee approved the protocol.

### Settings

According to the latest national census data, 55 ethnic minorities account for about 8.89% (125.47 million) of China's 1.4 billion people. One of their major settlements is in Yunnan province in rural southwest China. This survey was conducted in Nanmei township, an ethnic minority settlement in Linxiang District, Yunnan Province. 4,906 people lived in Nanmei township at the end of 2020, of which 4,160 (84.8%) were ethnic minorities, and the majority of the ethnic minority population was Lahu, with 3,547 people, accounting for 85.3% of the total ethnic minority population. The area is remote, economically backward, and lacks health resources. The per capita disposable income in 2020 is 10,530 yuan (Yunnan Province: 23,295 yuan), and there are 1.23 (Yunnan Province: 2.5) practicing physicians per 1,000 people. which is far below the average of Yunnan Province.

### Sampling

The following formula performed the sample size calculation:


$$\begin{array}{l} N=\frac{Z_{1-\alpha /2}^{2}\times \pi \left(1-\pi \right)}{\delta^{2}}\; \end{array}$$

(π: expected adequate KAP rate; δ: sampling error)

According to Zhang et al. (15), the adequate KAP rate of chronic diseases among ethnic minority populations in China was 47.9%. In this study, π = 0.48, $Z_{1-\alpha /2}^{2}=$ 1.96, δ = 0.1 π = 0.048, α = 0.05. We found that the required sample size was 435. To accommodate the design effect, the calculated sample size was multiplied by 1.8 for correction, alongside the 20% refusal rate, the final sample size of the survey was determined to be 870.

A systematic sampling method was used to select one out of every five households in 1,388 households in Nanmei Town. A total of 290 households were sampled, yielding 872 potential survey respondents, with 801 people actually completing the survey. Uniformly trained investigators will conduct household surveys of households determined at the sampling stage, each investigator equipped with an investigator's handbook for inquiries, and bring an interpreter with them when investigating ethnic minority participants. Han Chinese survey respondents and minority respondents who speak Chinese completed the survey in Chinese, and minority respondents who do not speak Chinese had their questions verbally translated by an interpreter to complete the survey.

### Development of the KAP questionnaire for common chronic diseases

We extracted a pool of items on knowledge, attitude, and practice (KAP) related to four common chronic diseases (hypertension, diabetes, obesity, and chronic obstructive pulmonary disease) from previous research (16–19) and then selected appropriate items from the pool to form the KAP questionnaire of common chronic diseases used in this study, and conducted a pre-survey (50 participants included).

Knowledge was measured by statements such as: “Overeating oily food regularly can cause high cholesterol,” “Smoking is a factor in causing chronic obstructive pulmonary disease (COPD),” and “Blood pressure has two values: systolic (high) and diastolic (low),” “Diabetes have far reaching effects on various body organs such as the eyes, nerves, feet, and kidneys,” and so on. Participants responded with discrete answers (True/False/Uncertain) and got 1 point for a correct answer; otherwise, no point. In addition, some questions were reverse coded, e.g., “Passive smoking does not cause chronic obstructive pulmonary disease.”

Attitudes were measured by the following statements. “You are willing to learn about healthy diet,” “You believe that systematic rehabilitation can slow the progression of COPD,” “You believe that high salt consumption can lead to high blood pressure,” “If you are considered a high-risk population for diabetes, you feel you should acquire knowledge about diabetes,” and so on. Participants selected “Agree” (1 point), “Not sure” (0 points), or “Disagree” (0 points) from the Likert items. Some statements were reverse coded, e.g., “Patients with COPD should not be receiving long-term oxygen therapy because of the potential for dependence.”

Practice was measured by the healthy lifestyle and practice standards proposed in the 66 health literacy items for Chinese residents published by the National Center for Health Education, including smoking (1 point), alcohol consumption (1 point), fruit and vegetable intake (2 points), fried food intake (1 point), sleep (1 point), physical activity (1 point), chronic diseases prevention and control (4 points).

Results from the pre-survey showed that the internal consistency of the subscales was adequate: 0.77 for knowledge (mean score: 17.74 ± 0.2.5 out of a total score of 24), 0.83 for attitude (mean score: 17.44 ± 2.1, out of a total score of 20), and 0.71 for practice (mean score: 5.76 ± 1.5 out of a total score of 11). The content validity of the questionnaire was rated by eight professionals with expertise in chronic disease prevention and control, and the overall content validity index (CVI) of the questionnaire was 0.83.

### Other study measures

The participants' age, sex, and nationality were obtained *via* residents' I.D. cards. Other information (education level, marital status, annual income, living with whom, provided by whom, and annual out-of-pocket medical expense) was collected *via* participants' reports. Chronic disease conditions were measured as if participants had been diagnosed with one of the four common chronic diseases or any another one.

### Statistical analysis

We firstly standardized each KAP subscale score; each score of the subscale was subtracted from the mean of the subscale scores and then divided by the root mean square of the subscale scores. Then, we performed a latent profile analysis of the standardized score to identify sub-populations of participants presenting distinct KAP profiles toward common chronic diseases with a robust estimator of nonnormality (robust maximum likelihood). We examined solutions with 1 to 6 distinct profiles and replicated each latent profile solution ten times, beginning at random starting values. We considered the best solution to be parsimonious, to have profiles with conceptual meaning, and to have the best fit (as indicated by the lowest Bayesian information criterion and integrated completed likelihood). Bootstrap Likelihood Ratio Test (BLRT) was conducted to observe if an increase in profiles increases fit. Although the Bayesian information criterion and integrated completed likelihood were slightly better for the 5-profile solution and BLRT showed no significant difference between the models of 5- and 6- profile, we chose the 3- profile solution as providing the most conceptually coherent description of participants' KAP features for common chronic diseases. We assigned participants to the latent profile group for which they had the highest membership probability. Descriptive statistics for demographic and clinical characteristics of participants are presented for the full sample and by latent profile groups. To explore potential differences in characteristics across latent profile groups, we performed multinomial logistic regression analyses. All *p*-values represent 2-sided hypothesis tests with a set significance level of 0.05. All analyses were conducted using R 4.1.2 (20).

## Results

We enrolled 801 participants after screening 872 participants regarding potential participation (91.85% participation rate). The most common reasons offered for declining to participate included “not interested” [31 (43.1% of nonparticipants)], “physical reasons” [9 (12.5%)], and “don't have enough time” [32 (44.4%)].

The majority of participants in our sample were ethnic minorities (61.9%), and the rest were Han Chinese (38.1%). The mean age of participants was 55 years-old. Thirty-six (4.49%) participants were with education level of high school and above, 110 (13.7%) were with education level of middle school, 347 (43.3%) were education level of primary school, and 308 (38.5%) were without formal education. The annual income of 321 (40.1%) participants was less than the per capita disposable income of Yunnan Province in 2020, 287 (35.8%) participants had an annual income within two times the per capita disposable income of Yunnan Province in 2020, and 193 (24.1%) participants had an annual income more than two times the per capita disposable income of Yunnan Province in 2020. 331 participants (41.9%) relied on others to provide for their living expenses. Two hundred forty-nine (31.1%) participants self-reported hypertension, 63 (7.87%) participants self-reported diabetes, and 483 (60.3%) participants self-reported no chronic diseases. In addition, six participants (0.75%) reported other chronic diseases such as ischemic stroke or rheumatoid arthritis.

**Figure 1:**
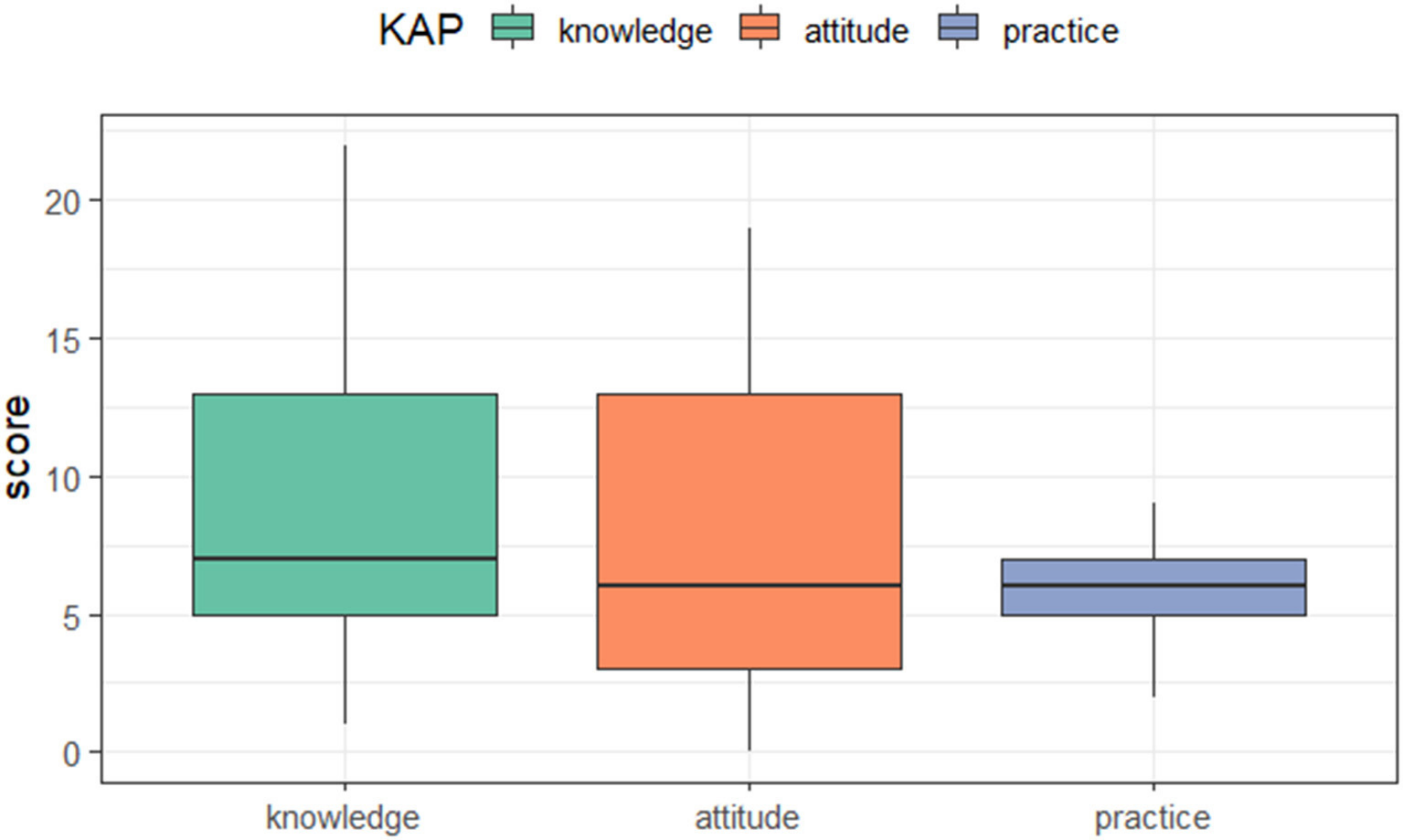
The distribution of KAP score.

The distribution of the KAP score was shown in Figure 1. Participants scored from 1 to 22 on the knowledge section (median: 7 points, IQR: 5–13), from 0 to 19 on the attitude section (median: 6 points, IQR: 3–13), and from 2 to 9 on the practice section (median: 6 points, IQR: 5–7).

### Common patterns of participants' KAP scores toward common chronic diseases

**Figure 2:**
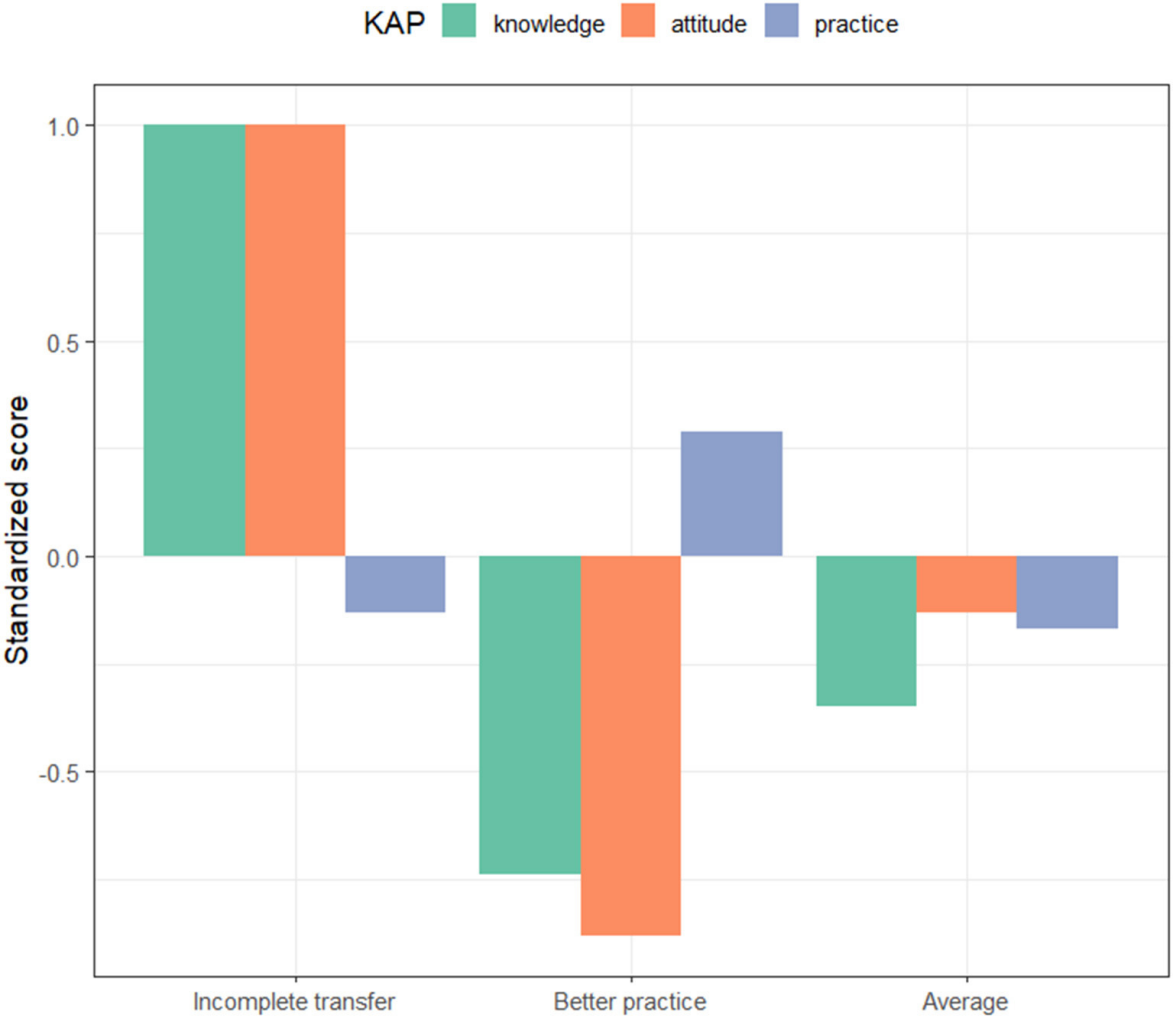
Participants' KAP profiles.

Three-profile solution was determined after the latent profile analysis: incomplete transfer [I.T.] (*n* = 215), better practice [B.P.] (*n* = 301), and average [A.V.] (*n* = 285). The propensity of the KAP score defines each profile and reveals its differences. The first profile (26.84%) was characterized by the highest levels of knowledge and attitudes toward common chronic diseases and below-average levels of practice, indicating that the transfer from knowledge and attitudes to practice was incomplete among participants in this subgroup and, therefore, was labeled as “incomplete transfer” (I.T). Participants in this group may be rich in chronic disease-related knowledge and have developed positive attitudes, but lack the ability to engage in appropriate health practices, perhaps with barriers to practice that are not easily overcome or that entail greater costs that offset the benefits of practice. The second profile (37.58%) was characterized by poor performance in terms of knowledge and attitudes toward common chronic diseases, but the highest level of practice, referred to as “better practice” (B.P.). Subjects in this group may not fully understand chronic disease-related knowledge or attitudes due to a low level of education, but are willing (or compelled) to improve their health practices as recommended by their physicians. Lastly, the third profile (35.59%) was reflected average knowledge, attitude, and practice toward common chronic diseases among three subgroups, which was labeled “average” (A.V.). These “average” participants' knowledge, attitudes, and practices toward common chronic diseases fell between the I.T. and B.P. groups, and they may have some level of knowledge and positive attitudes related to chronic diseases, but occasionally encounter obstacles in their practices (Figure 2).

### Characteristics of participants in distinct KAP latent profile groups

We examined whether the three KAP latent profile groups were associated with characteristics of participants (Table 1). Overall differences were found in a few characteristics (nation, education level, marriage status, living arrangement, annual family income, self-reported chronic disease, and annual out-of-pocket medical expense). Odds ratios were obtained after being adjusted by multinomial logistic regressions (Table 2).

**Table 1:** Participant characteristics for full sample and by KAP latent profile groups.

**Characteristics**	**All**	**Incomplete transfer**	**Better practice**	**Average**	***p*** **-Value**
	***N*** = **801**	***N*** = **215**	***N*** = **301**	***N*** = **285**	
**Sex:**					**0.262**
Male	382 (47.7%)	104 (48.4%)	133 (44.2%)	145 (50.9%)	
Female	419 (52.3%)	111 (51.6%)	168 (55.8%)	140 (49.1%)	
Age	55.1 (17.3)	56.8 (17.3)	53.5 (17.3)	55.5 (17.1)	0.095
**Nation:**					<0.001
Han	305 (38.1%)	129 (60.0%)	87 (28.9%)	89 (31.2%)	
Ethnic minorities	496 (61.9%)	86 (40.0%)	214 (71.1%)	196 (68.8%)	
**Education:**					<0.001
No formal education	308 (38.5%)	59 (27.4%)	116 (38.5%)	133 (46.7%)	
Primary school	347 (43.3%)	105 (48.8%)	142 (47.2%)	100 (35.1%)	
Middle school	110 (13.7%)	39 (18.1%)	37 (12.3%)	34 (11.9%)	
High school and above	36 (4.49%)	12 (5.58%)	6 (1.99%)	18 (6.32%)	
**Marriage:**					0.547
Unmarried	39 (4.87%)	10 (4.65%)	18 (5.98%)	11 (3.86%)	
Married	651 (81.3%)	173 (80.5%)	238 (79.1%)	240 (84.2%)	
Divorced	111 (13.9%)	32 (14.9%)	45 (15.0%)	34 (11.9%)	
**Living arrangement:**					<0.001
Alone	24 (3.00%)	7 (3.26%)	8 (2.66%)	9 (3.16%)	
With spouse	374 (46.7%)	64 (29.8%)	167 (55.5%)	143 (50.2%)	
With children	351 (43.8%)	128 (59.5%)	103 (34.2%)	120 (42.1%)	
With other relatives	52 (6.49%)	16 (7.44%)	23 (7.64%)	13 (4.56%)	
**Annual income:**					<0.001
Low	321 (40.1%)	68 (31.6%)	152 (50.5%)	101 (35.4%)	
Moderate	287 (35.8%)	81 (37.7%)	95 (31.6%)	111 (38.9%)	
High	193 (24.1%)	66 (30.7%)	54 (17.9%)	73 (25.6%)	
**Living expense:**					0.638
Self-supplied	470 (58.7%)	132 (61.4%)	174 (57.8%)	164 (57.5%)	
Supplied by others	331 (41.3%)	83 (38.6%)	127 (42.2%)	121 (42.5%)	
**Annual out-of-pocket expense:**					<0.001
Affordable	651 (81.3%)	196 (91.2%)	278 (92.4%)	177 (62.1%)	
High	125 (15.6%)	9 (4.19%)	21 (6.98%)	95 (33.3%)	
Catastrophic	25 (3.12%)	10 (4.65%)	2 (0.66%)	13 (4.56%)	
Self-reported hypertension	249 (31.1%)	100 (46.5%)	66 (21.9%)	83 (29.1%)	<0.001
Self-reported diabetes	63 (7.87%)	15 (6.98%)	37 (12.3%)	11 (3.86%)	0.001
Self-reported no chronic disease^a^	483 (60.3%)	102 (47.4%)	189 (62.8%)	192 (67.4%)	<0.001

^a^None of the participants reported COPD or obesity.

**Table 2:** Logistic regression analysis of differences in characteristics across KAP latent profile groups.

	**Incomplete transfer vs. Better practice**			**Incomplete transfer vs. Average**			**Average vs. Better practice**		
	**OR**	**95% CI**	***p*** **-Value**	**OR**	**95% CI**	***p*** **-Value**	**OR**	**95% CI**	***p*** **-Value**
Female sex	1.13	0.74–1.72	0.58	0.87	0.57–1.32	0.51	1.29	0.88–1.91	0.19
Age	0.98	0.96–1.00	0.03	0.99	0.97–1.01	0.19	0.99	0.98–1.01	0.34
Ethnic minorities	3.46	2.23–5.36	<0.01	2.53	1.63–3.93	<0.01	1.37	0.89–2.10	0.15
**Education**									
No formal education	1 (ref)	–	–	1 (ref)	–	–	1 (ref)	–	–
Primary school	0.84	0.50–1.40	0.50	0.52	0.31–0.87	0.01	1.62	1.02–2.57	0.04
Middle school	0.37	0.17–0.82	0.01	0.51	0.24–1.10	0.09	0.73	0.35–1.50	0.39
High school and above	0.14	0.04–0.49	<0.01	0.84	0.31–2.26	0.73	0.17	0.05–0.53	<0.01
**Marriage**									
Unmarried	1 (ref)	–	–	1 (ref)	–	–	1 (ref)	–	–
Married	0.34	0.10–1.23	0.10	0.49	0.13–1.92	0.31	0.70	0.19–2.65	0.60
Divorced	0.57	0.15–2.25	0.42	0.40	0.09–1.76	0.23	1.42	0.34–5.88	0.63
**Living arrangement**									
Alone	1 (ref)	–	–	1 (ref)	–	–	1 (ref)	–	–
With spouse	2.60	0.76–8.97	0.13	2.30	0.62–8.46	0.21	1.13	0.35–3.65	0.83
With children	0.87	0.26–2.91	0.82	1.67	0.47–6.02	0.43	0.52	0.16–1.66	0.27
With other relatives	0.87	0.20–3.76	0.85	0.54	0.11–2.57	0.44	1.61	0.36–7.31	0.53
**Annual income**									
Low	1 (ref)	–	–	1 (ref)	–	–	1 (ref)	–	–
Moderate	0.50	0.31–0.80	<0.01	0.59	0.36–0.96	0.03	0.85	0.54–1.34	0.49
High	0.37	0.21–0.64	<0.01	0.56	0.32–0.97	0.04	0.65	0.38–1.11	0.12
**Living expense**									
Self-supplied	1 (ref)	–	–	1 (ref)	–	–	1 (ref)	–	–
Supplied by others	1.26	0.76–2.09	0.36	1.06	0.64–1.77	0.81	1.19	0.75–1.87	0.46
**Annual out-of-pocket** **medical expense**									
Affordable	1 (ref)	–	–	1 (ref)	–	–	1 (ref)	–	–
High	1.13	0.47–2.74	0.78	11.84	5.42–25.87	<0.01	0.10	0.05–0.17	<0.01
Catastrophic	0.11	0.02–0.58	0.01	1.88	0.71–4.97	0.20	0.06	0.01–0.29	<0.01
Self-reported hypertension	0.25	0.11–0.53	<0.01	1.10	0.47–2.61	0.82	0.22	0.10–0.49	<0.01
Self-reported diabetes	2.06	0.96–4.43	0.06	0.50	0.19–1.28	0.15	4.16	1.79–9.69	<0.01
Self-reported no chronic diseases	0.73	0.35–1.50	0.39	2.68	1.17–6.15	0.02	0.27	0.13–0.56	<0.01

Participants with higher education level were less likely to be in B.P. (OR = 0.14, *p* < 0.01) or A.V. (OR = 0.84, *p* = 0.73) group than I.T. group. The I.T. group had the largest percentage participants who lived with their children (59.5%), and had high annual income (30.7%), most participants in this group were self-supplied (61.4%), nearly half reported having hypertension (46.5%). Ethnic minorities were more likely to be in B.P. group than I.T (OR = 3.46, *p* < 0.01) or A.V. group (OR = 1.37, *p* = 0.15). This group also had the largest proportions of participants who lived with spouse (55.5%), had low annual income (50.5%) and affordable annual out-of-pocket medical expense (92.4%). Participants who self-reported no chronic disease were more likely to be in the A.V. group than I.T. (OR = 2.68, *p* = 0.02) or B.P. group (OR = 0.27, *p* < 0.01). Also, like the B.P. group, ethnic minorities were the dominant population in the A.V. group (68.8%). Furthermore, the A.V. group contained the largest proportions of married participants (84.2%), participants with no formal education (46.7%), and high annual out-of-pocket medical expense (33.3%).

## Discussion

Management services for common chronic diseases have been incorporated into the national basic public health services, including individualized health education services. Through conversations with residents in ethnic minority areas, members of the general practitioner team and social workers generally felt a gulf to be crossed between these residents' health literacy and the available health education services. These analyses of residents' knowledge, attitudes, and practice about common chronic diseases may help understand their health needs and therefore influence how recipients and providers choose appropriate health education services. This choice may be interpreted by providers as relinquishing some of their health management responsibilities but is actually a prioritization of different needs (e.g., the I.T. profile, which requires behavioral management, and the B.P. profile, which lacks knowledge and beliefs). Without an adequate understanding of residents' health literacy and health needs, providers may be ill-prepared to understand residents' choices and help them manage their own health (21).

Research studies on health literacy levels of ethnic minorities have been conducted in Turkey, Sweden, and China (8, 22, 23), and these studies share the understanding that low levels of health literacy are evident in ethnic minorities; however, further individual differences lack relevant research work. This study refined the understanding of the health literacy of residents in ethnic minority areas as reflected in the focus of their KAP scores through the common chronic disease KAP questionnaire and subsequent latent profile analysis. We identified three groups of participants with similar KAP patterns (I.T., B.P., A.V.) and found that certain demographic and clinical characteristics appeared to be associated with group membership. Specifically, the I.T. group included more Han Chinese participants with higher annual incomes, suggesting that people of higher socioeconomic status may be more receptive to health knowledge but less able to translate that knowledge into practice. In contrast, the B.P. group included more minorities, less educated participants, fewer participants who suffered catastrophic medical costs, and more participants who self-reported having diabetes, suggesting that those who are more aware of their health conditions may be more concerned about improving their health practice, even if they are less knowledgeable and correspondingly less at risk for catastrophic medical expenses. In the case of the A.V. group, as the group description implies, participants in this group scored mediocre in all aspects, with demographic characteristics of being more ethnic minority, having lower education levels and lower annual income, and bearing more risk of higher medical expense compared to the I.T. group. There are no significant differences in demographic characteristics of the A.V. group compared to the B.P. group, except for higher education levels and medical expenses. Notably, the A.V. group had the highest self-reported rate of no chronic disease out of the three groups. These findings suggest that some minorities are likely to acquire knowledge from existing health education services, but the translation from health knowledge to health behaviors remains problematic. The differences in participant scores highlight important differences in knowledge, attitudes, and practice concerning common chronic diseases among participants and indicate their essential health needs. The KAP latent profile groups underscore a key point: there is no single correct pattern of health education services for populations with different health needs.

Through previous research (24), we have learned that health inequities exist in health education and health promotion. Ethnic minorities are disadvantaged in many countries regarding accessibility and utilization of health education services (25–27). The question is whether this disadvantage is followed by different unsatisfied health needs, which is confirmed by the results of the present study, in which we selected a typical ethnic minority area as the study site. After analyzing residents' knowledge, attitudes, and practice regarding common chronic diseases, we identified three KAP latent profile groups and the different health needs underlying them. First, there is a clear knowledge-practice gap in the I.T. group, and applying the behavioral economics framework to the field of health education can reveal potential barriers to translating knowledge into practice and entry points for interventions by clinicians and public health professionals (28); furthermore, the development of mobile health technologies, especially the availability of wearable devices and 5G/6G wireless technologies, has made real-time monitoring and telehealth possible (29), facilitating the implementation of behavioral interventions. Second, we argue that the same ethnic minorities still have different health needs; for example, the B.P. group may need more culturally sensitive health knowledge, while the A.V. group has a more substantial need to address the translation of knowledge to practice. These findings may provide novel ideas for future health education work: precise identification of needs followed by corresponding precise interventions (30).

The purpose of this study was not to reaffirm the inadequate health literacy among ethnic minorities; the knowledge, attitudes, and practice about common chronic diseases that we investigated are important components of health literacy but do not fully represent health literacy that is still evolving (31). Instead, the findings reported here present an advance in the study of health literacy among ethnic minorities; the health needs of ethnic minorities should be well understood before we conduct research on health literacy interventions. Some evidence suggests that model-based health literacy assessments or interventions can provide a better picture of these needs (32, 33).

A limitation of this study is that the differences in KAP for chronic diseases among different ethnic minorities are not adequately discussed. This present study focuses on the differences between chronic disease knowledge, attitudes, and practice between Han and ethnic minorities; however, as we mentioned, there are 55 different ethnic minorities in China who may have different levels of acceptance of generalized health education due to their customs, language and script, degree of Hanization (assimilated by Han Chinese), etc., and thus differences between KAP for chronic diseases. However, in our study population, the ethnic minority study population was predominantly Lahu (447 of 496), so this variation in chronic disease KAP among different ethnic minorities was not influential on the overall results. In addition, our search of previous literature revealed some commonalities across ethnic minorities in Yunnan: high illiteracy rates, low awareness of their health concerns, and high tobacco and alcohol consumption (34, 35). Another limitation is that we used self-reported prevalence of chronic diseases. Among the four common chronic diseases surveyed, no one reported prevalence of two chronic diseases (chronic obstructive pulmonary disease and obesity), which is inconsistent with the facts we learned from local CDC and hospitals, and the existence of reporting bias should not be ignored. We will consider this in the intervention design phase of the upcoming health literacy intervention study.

## Conclusion

Having a more specific and nuanced understanding of ethnic minority health literacy allows providers to conduct more effective health education with their recipients. Given the differences in health needs, this will naturally lead to different offerings. Providers can do more to encourage recipients to be proactive in managing their own health and to take responsibility for maintaining it. Then, professionals use their specialization to help recipients make health decisions, rather than mandating things or instilling expertise that is difficult to grasp. This form of health education activity builds better relationships between providers and recipients and permits professionals to engage in this work in an empowering way, thereby improving health inequities.

## Data availability statement

The raw data supporting the conclusions of this article will be made available by the authors, without undue reservation.

## Ethics statement

The studies involving human participants were reviewed and approved by Lincang Second People's Hospital. The patients/participants provided their written informed consent to participate in this study.

## Author contributions

HH, YX, YS, and YL designed the study. YS and YL cleaned and processed the data. HH wrote the manuscript. YX and CL revised the manuscript. All authors contributed to the subsequent drafts, reviewed, and endorsed the final submission.

## Conflict of interest

The authors declare that the research was conducted in the absence of any commercial or financial relationships that could be construed as a potential conflict of interest.

## Publisher's note

All claims expressed in this article are solely those of the authors and do not necessarily represent those of their affiliated organizations, or those of the publisher, the editors and the reviewers. Any product that may be evaluated in this article, or claim that may be made by its manufacturer, is not guaranteed or endorsed by the publisher.
